# Pressure Ulcer in Norway—A Snapshot of Pressure Ulcer Occurrence across Various Care Sites and Recommendations for Improved Preventive Care

**DOI:** 10.3390/healthcare3020417

**Published:** 2015-06-09

**Authors:** Edda Johansen, Linda N. Bakken, Zena Moore

**Affiliations:** 1 Department of Nursing Science, Faculty of Health Sciences, Buskerud and Vestfold University College, Grønland 58, Drammen 3045, Norway; E-Mail: linda.bakken@hbv.no; 2 School of Nursing and Midwifery, Royal College of Surgeons, 123 St Stephen’s Green, Dublin 2, Ireland; E-Mail: zmoore@rcsi.ie

**Keywords:** pressure ulcer, prevalence, prevention, Norway

## Abstract

Pressure ulcers (PU) are common in all care settings, although most ulcers are preventable. Much evidence exists on Hospital Acquired Pressure Ulcers (HAPU), however, few studies describe PU in community care. From a Norwegian perspective, little is known about pressure ulcer prevalence and prevention strategies across the variety of healthcare sectors. Therefore, this study explored PU prevalence and preventive care in home care, nursing homes and hospitals. Seventeen postgraduate wound care students collected data. A data collection instrument by Jordan O’Brien and Cowman was used together with an online forum in which students described how to improve practice to reduce PU incidence. This study showed that pressure ulcers are a problem across all care settings in Norway; however, nursing homes had the highest proportion of at risk patients and the highest prevalence. By implementing the care bundle provided by the Patient Safety Programme across all care settings, increasing staff competency and make sure that access to appropriate equipment for beds and chairs is readily available, a structured and evidence based approach to prevention could be ensured.

## 1. Introduction

A pressure ulcer is defined as “localized injury to the skin and/or underlying tissue usually over a bony prominence, resulting from sustained pressure (including pressure associated with shear). A number of contributing or confounding factors are also associated with pressure ulcers; the primary of which is impaired mobility” [1]. As indicated by the definition, pressure ulcers commonly occur in individuals who have activity or mobility problems and as such are exposed to prolonged periods of exposure to sustained pressure/shear forces [2]. Thus, the elderly, those with spinal cord injury, and those who are sedated following trauma or surgery are particularly at risk of developing pressure ulcers [3,4,5,6]. However, any person of any age could potentially develop a pressure ulcer if they were exposed to the causative factors of sustained unrelieved pressure and shear forces.

The presence of pressure ulcers among individuals in the care of health professionals is often used as an indicator of the quality of healthcare provided [7]. Therefore, in order to place the problem of pressure ulcers into context, the number of pressure ulcers within a given clinical care setting is measured and reported as prevalence or incidence figures [1]. Prevalence is a determination of the number of people with an existing pressure ulcer at a given point in time, whereas incidence is a determination of the number of people that develop a new pressure ulcer over a given time period [8].

A recent integrative review noted mean prevalence rates of 8.9% in Iceland (*n* = 1 study), 17% in Norway (*n* = 3 studies), 16% in Ireland (*n* = 6 studies), 15% in Denmark (*n* = 5 studies) and 25% in Sweden (*n* = 17 studies) [5]. These studies were conducted in acute-care (*n* = 23); long stay (*n* = 5); hospice (*n* = 1), community care (*n* = 2) and in a center for rare diseases (*n* = 1), supporting the argument that limited evidence exist pertaining to knowledge on pressure ulcer prevalence within the primary care setting [9]. Incidence figures also vary across countries, for example, a single incidence study from Norway noted a figure of 16.4%, whereas mean incidence in Denmark was 1.8% (*n* = 2 studies), 11% in Ireland (*n* = 4 studies) and 20% in Sweden (*n* = 12 studies) [5]. Prevalence and incidence figures are consistently highest in acute care and hospice settings and lowest in elderly care settings [5,10]. Indeed, not all studies clearly delineated between categories of PU, furthermore, different classification systems were used, including self-developed classification systems, the EPUAP pressure ulcer classification system, the EPUAP/National Pressure Ulcer Advisory Panel (NPUAP) guidelines and sometimes none at all [5].

Variations in pressure ulcer occurrence across countries may relate to the huge differences found in hospital and municipal services in Europe [11]. In Norway, for example, health care services are provided at the lowest feasible care level [12], resulting in early discharge of patients from hospitals. The number of hospital beds are therefore reduced and the average length of stay in Norwegian hospitals is 4.1 days [13]. The early discharge of patients from hospitals has meant that nursing home populations are old and frail with significant functional impairment and multiple active diseases [14].

Prevalence and incidence studies have the potential to identify areas of concern and target areas requiring improvements to reduce a problem. However, although a great number of prevalence and incidence studies do exist, the prevalence remains unchanged [10], indicating that patients at risk are not necessarily receiving appropriate preventive care. Adequate pressure ulcer prevention involves simple bedside initiatives—risk assessment, skin inspection and skin care, use of appropriate surfaces in beds/chairs, repositioning, nutritional assessments and supply of supplements, and interventions as necessary [1,15]. However, a lack of access to appropriate prevention equipment adversely affects preventive work [16,17,18]. Similarly, insufficient knowledge and a lack of interest also hamper pressure ulcer prevention [19,20]. A lack of reduction in pressure ulcers is worrying because pressure ulcers have a significant impact on health related quality of life, with all of the activities of daily living adversely affected, often resulting in anxiety, depression [21] and pain [22].

In an economically constrained health service, money spent on pressure ulcers is a concern, as it is suggested that many pressure ulcers can be avoided with appropriate risk assessment and use of interventions targeted at combating this risk [23]. Despite this premise, it is estimated that approximately 4% of the annual healthcare budget is being spent on pressure ulcers, with nursing time accounting for 41% to 96% of these costs [24,25]. Furthermore, pressure ulcers have been shown to increase length of hospital stay, readmission and mortality rates [26], and add considerably to the cost of an episode of hospital care [27]. Therefore, the aims of this study were to carry out a prevalence study in different care sites in Norway, to identify areas of concern and from this to determine how best to improve preventive practice.

## 2. Methods

### 2.1. Study Design and Settings

A cross-sectional study was undertaken with data collected from different hospitals and community care sites in Norway (*n* = 14). Seventeen postgraduate wound care students collected the data on one day in November 2014. They were all registered nurses with a minimum of 2 years of clinical experience. The students came from widespread geographical areas throughout Norway and worked in hospitals, home care and nursing homes, in both urban and rural areas. Six students carried out the study in pairs, whereas 11 students carried out the study individually, leaving the study with information from 14 care sites. They all used a modified data collection instrument by Jordan O Brien and Cowman [28] to investigate pressure ulcer prevalence, risk status and surfaces in patients’ beds and chairs. Fourteen students investigated pressure ulcers within their own organization, whereas 3 students, those who worked in pairs, carried out data collection with a fellow student in his or her workplace. Altogether, 149 patients aged 18 and older, volunteered for the study and their skin was inspected. As 3 forms had substantial missing data, they were excluded, leaving 146 records to be included in the analysis.

### 2.2. Ethical Considerations

Permission to carry out the work was obtained individually by the students (as relevant) from the local governance authority in each study site. Patients and/or relatives were informed verbally about the study, how it related to students course work and that participation was voluntary. Oral consent was obtained and skin inspection was carried out on those who volunteered. To avoid extra strain on participants, skin inspection was made in conjunction with regular visits, for example when patients received help with personal hygiene.

### 2.3. Training

All 17 students that participated in the survey undertook an e-learning course on PU staging, which also consisted of a multiple-choice test involving categorization of pressure ulcers. In class, they learned how to fill in the data collection instrument and how to carry out the study. They were also provided with information on epidemiology, pressure ulcer prevention, the content of the Norwegian Patient Safety Programme for pressure ulcer prevention, and how the Patient Safety Programme has been implemented in an urban Norwegian hospital.

### 2.4. Data Collection Tool

The instrument captured data on care facility, gender, age group, pressure ulcer risk (a Norwegian version of the Braden score), continence, location and category of the deepest pressure ulcer and support surfaces in bed and chairs. The Braden scale consists of six subscales: sensory perception, mobility, moisture, nutrition, activity and friction/shear. Five of the six subscales are scored from 1 to 4, whereas friction/shear is scored 1–3. The six subscales are summarized and the total score ranges from 6 to 23, with lower scores indicating higher risk of pressure ulcers. The scores on the Norwegian version of the Braden scale is categorized into: very high risk, 9 or less; High risk, 10–12; Moderate risk, 13–14; Mild Risk, 15–18; and No risk, 19–23. The Norwegian version of Braden follows Bergstrom, *et al.* [29], which recommend the cut-off to be set at 18.

### 2.5. Data Analysis

Analysis was carried out using IBM SPSS statistics version 22 (SPSS Inc., Chicago, IL, USA). Chi-square test (χ^2^) and Fisher’s Exact test were used to assess the relationships between variables with *p* set at <0.05. A content analysis was made from students’ documentation describing how to improve practice to reduce PU incidence.

## 3. Results

### 3.1. Age, Gender and Care Site

Participant (*n* = 146) came from 14 different care sites: three nursing homes, six home care districts, one institution hosting patients rotating between home care and institutional care on a regular basis (alternating patients) (HC/NH) and four hospitals (see Table 1). The sample consisted of 80 patients from the community and 66 from hospitals, a total of 64 men and 79 women.

It was found that 97% of nursing home patients were 80 years and older. In the home care population, 67% were older than 80, with the remaining patients being within all age categories. In hospital care sites, patients in all age groups were represented, with no age group overrepresented.

**Table 1:** Population by care site.

Community Care Sites % (*n*)				Hospitals % (*n*)		
55 (80)				45 (66)		
Nursing home	Home care	HC/NH		Surgical	Orthopedic	Medical
39 (31)	39 (31)	22 (18)		39 (26)	26 (17)	35 (23)

### 3.2. Number and Grades of Pressure Ulcers

Thirty-five individuals were identified with pressure ulcers, yielding a prevalence of 24%. The prevalence in hospitals was 18% (*n* = 12). In nursing homes, the prevalence was 48% (*n* = 15); in home care, 16% (*n* = 5); and in alternating patients, 17% (*n* = 3). In nursing homes and hospitals, category 1 (nonblanchable erythema) comprised most pressure ulcers, whereas in home care the majority was category 3 (full thickness skin loss) and 4 (full thickness tissue loss). In alternating patients, the most severe pressure ulcer found was category 2 (partial thickness skin loss) (see Table 2).

**Table 2:** Most severe pressure categories by care sites.

Community Care Sites				Hospitals	Total
Site	Nursing home	Home care	HC/NH	Acute Care	
	Pressure Ulcer Grade % (*n*)				
Stage 1	26 (9)	0 (0)	3 (1)	17 (6)	46 (16)
Stage 2	14 (5)	0 (0)	6 (2)	6 (2)	26 (9)
Stage 3	0 (0)	2 (1)	0 (0)	6 (2)	8 (3)
Stage 4	3 (1)	6 (2)	0 (0)	6 (2)	15 (5)
Unknown	0 (0)	6 (2)	0 (0)	0 (0)	6 (2)
Total	43 (15)	14 (5)	9 (3)	34 (12)	100 (35)

Of those with pressure ulcers, 44 ulcers were registered for location, showing that the sacrum, ischial tuberosity and heels were the most common sites. Other ulcers were located on the feet, ears and back (see Table 3).

**Table 3:** Pressure ulcer location.

Community Care Sites				Hospital	Total
Site	Nursing home	Home care	HC/NH	Acute	
	Pressure Ulcer Location % (*n*)				
Heels	7 (3)	0 (0)	4 (2)	18 (8)	29 (13)
Ischia	14 (6)	7 (3)	0 (0)	0 (0)	21 (9)
Sacrum	11 (5)	0 (0)	4 (2)	16 (7)	31 (14)
Other	7 (3)	4 (2)	0 (0)	7 (3)	18 (8)
Total	39 (17)	11 (5)	8 (4)	41 (18)	100 (44)

### 3.3. Risk of Developing Pressure Ulcers

Of the 146 patients, five were not risk assessed, leaving the study with 141 patients that were risk assessed by the students as a part of the study with the Braden scale. In hospitals, 32% (*n* = 21) were at risk, in nursing homes 62% (*n* = 18) were at risk, in homecare, 31% (*n* = 9) were at risk and 28% (*n* = 5) of the alternating patients were at risk of developing pressure ulcers. There was a statistically significant association between having a pressure ulcer and being at risk using Braden (cut-off 18) (*p* < 0.001). See Table 4 for level of risk in different care sites according to the Braden scale.

**Table 4:** Pressure ulcer risk by Braden categories.

Community care sites				Hospital
**Care facility**	**Nursing home**	**Home care**	**HC/NH**	**Acute**
Risk assessed *n* (%)	29 (20)	29 (20)	18 (13)	65 (46)
**Risk Category % (*n*)**				
No risk (19–23)	38 (11)	69 (20)	72 (13)	68 (44)
Mild risk (15–18)	34 (10)	21 (6)	28 (5)	20 (13)
Moderate risk (13–14)	17 (5)	10 (3)	0 (0)	6 (4)
High risk (9 or less)	10 (3)	0 (0)	0 (0)	6 (4)

### 3.4. Support Surfaces in Bed and Chair

In nursing homes, 29 of the mattresses were inspected, of which 83% (*n* = 24) were deemed to be suited for use with at risk patients (for example cubical foam, viscoelastic overlay or alternating mattresses). However, the beds of at risk patients were not necessarily allocated appropriate mattresses, conversely, some patients assessed as not being at risk were supplied mattresses suited for at risk patients (*p* = 0.6). In homecare, it was difficult to categorize mattresses and cushions from the data collection sheet, as people had a wide variety of beds and chairs in use at home. However, none of the patients at home had an alternating mattress.

Of the mattresses used within the hospital setting, 61 were inspected, showing that 61% (*n* = 37) were compact foam. Thirty-nine percent (*n* = 24) of the mattresses for at risk patients were split foam, viscoelastic foam, air mattresses and alternating mattresses. In hospitals, there was a significant association between pressure ulcer risk and allocation of underlay in bed (*p* < 0.05), although some at risk patients 35% (*n* = 7) were nursed on a compact foam surface.

In hospitals, 43 chair cushions were assessed, of which 7% (*n* = 3) were made of more than 5 cm compact foam. No statistically significant association was found between patient pressure ulcer risk and type of cushion offered to hospital patients (*p* = 0.21). In nursing homes, 28 chairs were assessed, of which 54% (*n* = 15) were appropriate for at risk patients (for example air cells). There was a statistically significant association between risk and allocation of cushions to patients in nursing homes (*p* < 0.001).

### 3.5. Initiatives to Reduce Pressure Ulcer Incidence

Three overarching themes where identified in how students would reduce pressure ulcers within their own care setting: (1) implement the care bundle launched by the Norwegian Patient Safety Programme; (2) increase competency among staff; and (3) offer interdisciplinary care. A minority of students mentioned the need for improved competence among patients and the involvement of leaders to improve preventive care.

## 4. Discussion

Although this study only provides a snapshot of the problem of pressure ulcers across different care sites in Norway, the results indicate that pressure ulcers constitute an unnecessary burden on patients and as such it can also be concluded that these wounds contribute to a financial drain on both hospitals and community care. However, with increased competency, interdisciplinary care and evidence based care bundles, pressure ulcer prevention could improve.

### 4.1. Community Care Site

This study found that nursing home residents were particularly vulnerable to pressure ulcer development with 62% being at risk, compared to 31% in home care, 28% among alternating patients and 32% in hospitals (Braden cut-off 18). In a study from Belgian nursing homes, 32.4% were at risk using Braden (cut-off 17) [19] and another study from long-term care found that 77% were at low or no risk using Braden (cut-off 18) [4]. A study based in home care found that 66.7% of patients were at risk using the Waterlow risk assessment tool [30], however in their study only 35.7% were actually risk assessed, leaving it difficult to conclude about risk in this homecare population.

#### 4.1.1. Nursing Homes

The Norwegian strategies aiming for care at the lowest feasible level [12], combined with extended life expectancy, has created a nursing home population that is old, and frail with severe functional impairment and incidence of active diseases [14]. Although only a relatively small sample of patients was included in this study, results indicate that nursing homes patients in Norway are at a particular risk when compared to patients at home or in hospitals. In order to target appropriate prevention, nursing homes need evidence-based care bundles and staff with appropriate competency to implement the care bundle correctly. According to this study, although nursing homes had access to appropriate pressure re-distributing mattresses, at risk patients were being nursed on compact foam and non-risk patients on mattresses suited for at risk patients. Furthermore, it was identified that the care bundle for pressure ulcer prevention provided by the National Patient Safety Programme, was not well known among staff. The reason for this is that, unlike hospitals, adoption of the recommendations from the National Patient Safety Programme is voluntary for municipalities.

In nursing home patients, pressure ulcers were mainly located on ischia tuberosity, sacrum and heels and occurred in 48.4% of the population. The prevalence found in this study was considerably higher than in previous studies showing 9% in long-term care [4], 20.8% in nursing homes [19], and 18.2% in hospital settings. This study, therefore, diverges from previous studies that refer to lower pressure ulcer prevalence in long-term compared to hospitals [7,31]. This study involved a high proportion of at risk patients from nursing homes, however, high proportion of risk patients does not automatically mean that there should be a corresponding high pressure ulcer prevalence because with appropriate risk assessment and preventive care, most pressure ulcers should be avoided [23]. Therefore, the volume of risk patients and concurrent pressure ulcers in this study should be of concern, as it may have arisen due to a lack of appropriate preventive care.

#### 4.1.2. Home Care

In home care, although only mild or moderate risk was identified in 31% of the population, 16.1% had pressure ulcers and, of those registered, all were category 3 or 4 ulcers. Furthermore, most were located at ischia tuberosity, indication that prevention in chair or in the seated position had been insufficient. However, as this was a prevalence study, including all pressure ulcers present at the time of assessment, some of the ulcers may not necessary have developed in the home care setting. Asimus and Li [30] found that 28.2% of all pressure ulcers among homecare patients were hospital acquired and in Norway, where patients are transferred to different care levels according to needs, therefore it could be difficult to track where the ulcers actually arise. Demarré, Vanderwee, Defloor, Verhaeghe, Schoonhoven and Beeckman [19] found that prevention when seated was particularly poor in nursing homes, and that it could relate to a lack of appropriate pressure-redistributing cushions or inadequate repositioning while seated. Unfortunately, for those patients with category 3 and 4 ulcers at home, type of underlay in use on the chair was not registered, leaving it impossible to know whether appropriate pressure-redistributing cushions were available.

An important finding from home care was that no alternating mattress was in use, even though patients had category 3 and 4 ulcers. This is in keeping with Asimus and Li [30] who found that only a small percentage of high and very high-risk patients at home had an alternating air mattress (3.5%). According to European Pressure Ulcer Advisory Panel, National Pressure Ulcer Advisory Panel and Pan Pacific Pressure Injury Alliance [1], it is difficult to know exactly what type of mattress patients with category 3 and 4 ulcers should have, but an active support surface should be provided when frequent manual repositioning is challenging. In Norway, patients at home that are at risk or have a pressure ulcer, can be supplied a pressure redistributing mattress or cushion free-of-charge by the social welfare system (NAV). For those patients who wish to get redistributing underlays free-of-charge, access could be hampered by the time it takes to supply the right equipment. This supports Asimus and Li [30] who found a gap in evidence-based homecare practice because of the speed and lack of access to pressure redistributing devices. This study found that to overcome the delay in access to appropriate equipment, an increased interdisciplinary approach to pressure ulcer prevention was necessary. This is because physiotherapist and occupational therapists are responsible for ordering redistributing underlay to patients at home. Although an increased interdisciplinary approach could benefit patients, it could be argued that if an evidence-based algorithm or flow-chart could safely outline what type of underlay patients should have, patients themselves, informal and formal caregivers should be able to order the correct underlay without any delay. Indeed, to allow patients, informal caregivers and any caregiver to order the right equipment, existing routines, locally and within the social welfare system (NAV), need considerable revisions.

Although underlay in beds and chairs are important for pressure ulcer prevention, there is a risk that other important aspects of pressure ulcer prevention are ignored when appropriate mattresses are in place [32]. Therefore, although patients are provided with appropriate mattresses or cushions, risk assessment, skin inspection, repositioning and activity, nutritional screening, involvement of patients and caregivers and documentation must be provided [1,15]. According to European Pressure Ulcer Advisory Panel, National Pressure Ulcer Advisory Panel and Pan Pacific Pressure Injury Alliance [1], mobility and activity limitations is a precursor for pressure ulcer development and without mobility and activity limitations, other risk factors are not primarily important. Limited activity or immobility is therefore common in patients with an existing pressure ulcer [4,30,33,34]. For patients who live alone with limited or no access to help from informal caregivers, it could be challenging to get sufficient repositioning. In this study, patients at home had severe ulcers over the ischia tuberosity, indicating a problem with seating and repositioning. Homecare patients are therefore in a unique position compared to patients who dwell in institutions, because they cannot as readily be provided with repositioning and activity as and when it is needed.

#### 4.1.3. Competency in Community Care

In Norway, RN, assistant nurses and assistants care for people in community care [35] and the proportion of RN to assistant nurses and assistants is low. Because of their different educational level, knowledge of pressure ulcer prevention may vary. However, this study found that independent of education level, staff did not necessary have sufficient competency to prevent pressure ulcers. These findings coincide with Demarrè *et al.* [19] who found no difference in knowledge level on pressure ulcer prevention between nurses and nurse assistant. According to Sving*, et al.* [36], assistant nurses, rather than nurses, are more likely to be given responsibility for pressure ulcer prevention. As the care bundle provided by the National Patient Safety Programme offers a simple and evidence-based approach to pressure ulcer prevention, it should successfully support any care delivery, independent of educational level. Although the community care sector is not obliged to implement the care bundle as such, this study revealed that municipalities would benefit from implementing it to yield a structured and systematic approach to pressure ulcer prevention regardless of competency.

### 4.2. Hospital Care Sites

The pressure ulcer prevalence found across hospital care sites in this study, coincide with previous studies [7,37] and so does the location of ulcers, with pressure ulcers occurring primarily on heels and sacrum [4,34,37,38]. Although there seems to be little new information regarding pressure ulcers in hospitals in this study, an interesting finding was that a significant association was found between risk and allocation of underlay in bed. Previous studies based in Norwegian hospitals found that appropriate mattresses were not always provided to at risk patients [16,18,37] and in some hospitals, base mattresses were old and worn [18,39]. Therefore, this study may indicate that pressure ulcer prevention and access to appropriate mattresses may have improved after the Patient Safety Programme was launched in 2011.

## 5. Limitations

There are several limitations to consider in this study. First, the wide range of care site and limited sample could influence the findings. Second, the use of several students, with limited research experience, could also have influenced on the quality of the data and study findings; however, these students have extensive clinical experience and would have most likely enriched the study. An important experience from this study was how the data sheet did not suit the home care setting in relation to mattresses and cushions. Another important factor to consider is that home care patients may have been visited because of their need for wound care. This study excluded an investigation of patients’ records and initiatives outlined in care plans and therefore does not give a picture of all those initiatives made for prevention; however, as documentation of preventive initiatives has proven to be poor in previous studies, lack of exploration of documentation in this instance is unlikely to have influenced findings adversely.

## 6. Conclusions

This study found that pressure ulcers constitute a considerable problem across all care sites; however, nursing homes were found to have the highest proportion of at risk patients and the highest prevalence figures. Overall, pressure ulcer prevention was hampered by limited competency among staff, as evidence by the actual care provided, and a lack of access to appropriate equipment for beds and chairs. The gap in evidence-based practice seems to be less in the hospital care sites because they have implemented the care bundle provided by the Patient Safety Programme. However, hospitals are still short of appropriate cushions and mattresses for at risk patients. Although the municipalities are not obliged to implement the care bundle for prevention, this study suggests that it should guide practice because it can provide a structured and evidence based approach to pressure ulcer prevention.
